# The Role of Early Child Nutrition in Pulmonary Hypertension—A Narrative Review

**DOI:** 10.3390/children11111307

**Published:** 2024-10-29

**Authors:** Alina-Costina Luca, Cristina Stoica, Cosmin Diaconescu, Elena Țarcă, Solange Tamara Roșu, Lăcrămioara Ionela Butnariu, Bogdan Aurelian Stana, Bogdan Gafton, Antoanela Curici, Eduard Vasile Roșu, Dana Elena Mîndru

**Affiliations:** 1 Department of Pediatrics, Faculty of Medicine, “Grigore T. Popa” University of Medicine and Pharmacy, RO-700115 Iasi, Romania; alina.luca@umfiasi.ro (A.-C.L.); diaconescu.cosmin@email.umfiasi.ro (C.D.); aurelian.stana@umfiasi.ro (B.A.S.); eduard.rosu@umfiasi.ro (E.V.R.); mindru.dana@umfiasi.ro (D.E.M.); 2 The Emergency Hospital for Children “Sfanta Maria”, RO-700309 Iasi, Romania; stoica.cristina@email.umfiasi.ro; 3 Department of Surgery II—Pediatric Surgery, Faculty of Medicine, “Grigore T. Popa” University of Medicine and Pharmacy, RO-700115 Iasi, Romania; 4 Department of Nursing, Faculty of Medicine, “Grigore T. Popa” University of Medicine and Pharmacy, RO-700115 Iasi, Romania; solange.rosu@umfiasi.ro; 5 Department of Medical Genetics, Faculty of Medicine, “Grigore T. Popa” University of Medicine and Pharmacy, RO-700115 Iasi, Romania; ionela.butnariu@umfiasi.ro; 6 Department of Oncology, Faculty of Medicine, “Grigore T. Popa” University of Medicine and Pharmacy, RO-700115 Iasi, Romania; bogdan.gafton@umfiasi.ro; 7 Department of Cellular and Molecular Biology and Histology, ‘’Carol Davila’’ University of Medicine and Pharmacy, RO-050474 Bucharest, Romania; antoanela.curici@umfcd.ro

**Keywords:** pulmonary hypertension, nutrition, malnutrition, resveratrol, microbiome, vitamin

## Abstract

Pulmonary hypertension is a complex condition that has distinct characteristics in pediatric populations. This review explores the important role of early childhood nutrition in the growth, progression, and management of pediatric pulmonary hypertension. Nutritional deficiencies, including those of vitamins C, D, and iron, are closely linked to worse outcomes in children with this disease, emphasizing the importance of early intervention to prevent malnutrition and promote growth. Emerging research revealed that promising nutrients like resveratrol, along with modulation of the gut and respiratory microbiomes, may offer therapeutic advances for managing pulmonary hypertension. However, the current literature is limited by a lack of pediatric-specific studies, with much of the data extrapolated from adult populations and animal models, especially rats. This review highlights the need for targeted research to develop effective nutritional interventions aimed at improving outcomes for pediatric patients.

## 1. Introduction

Pulmonary hypertension (PH) is a complex pathology characterized by elevated pressure in the pulmonary artery that leads to dysfunction of the right ventricle with progressive right heart failure. The pathophysiology of pulmonary arterial hypertension (PAH) is distinguished by diminished generation of vasodilators, including prostacyclin and nitric oxide (NO), alongside the overexpression of vascular constrictors such as endothelin-1 and tromboxane. Consequently, most recent medications for PAH target these routes [1].

The defining factor for pulmonary hypertension is an increased mPAP over 20 mmHg, using right heart catheterization, in both adults and children [2]. There are multiple differences between children and adults. While both have increased pulmonary artery pressure, pediatric patients tend to be less likely to exhibit heart failure and edema, although they exhibit more medial hypertrophy when first examined [3]. Children with this illness have unique characteristics, but they participate in significantly fewer trials than adults (*p* < 0.01) and most of them are in developed countries [4].

The Evian classification of pulmonary hypertension (PH) diseases is structured into five categories, based on specific therapeutic interventions to address the underlying causes. These categories are (1) primary pulmonary hypertension (PPH), (2) pulmonary venous hypertension, (3) pulmonary hypertension associated with respiratory diseases or hypoxemia, (4) pulmonary hypertension caused by thrombotic or embolic diseases, and (5) pulmonary hypertension caused by conditions affecting pulmonary vascularization. Each category includes subsets that highlight different causes and affected areas [5].

The primary forms of pulmonary hypertension that may appear in the pediatric population include persistent pulmonary hypertension of the newborn (PPHN), congenital heart defects, developmental lung disorders, and idiopathic pulmonary arterial hypertension (IPAH) [6].

The clinical manifestations of pulmonary hypertension (PH) typically result from a discrepancy between vasoconstrictor and vasodilator effects within the pulmonary blood vessels, leading to increased pulmonary vascular resistance (PVR). This increase in PVR raises the afterload on the right ventricle, ultimately causing right ventricular insufficiency. PH can develop through three noticeable mechanisms: pulmonary blood vessel constriction, which may respond to therapy using vasodilatators; blood vessel remodeling, characterized by thickening of the media and adventitia in affected vessels; and reduced angiogenesis, leading to the trimming of the pulmonary vascular network [1,6].

Severe and persistent pulmonary hypertension is a major element facilitating early mortality, particularly in infants with severe congenital heart disease, highlighting the recognized need for extracorporeal membrane oxygenation (ECMO) and extended care in neonatal intensive care units. The increased prevalence of pulmonary hypertension in premature infants, along with its correlation with adverse short- and long-term outcomes, underscores important implications for clinical care and trials research and the importance of involving preterm infants [7]. Preterm infants have distinct nutritional needs due to their accelerated growth and increased metabolic demands. Adequate nutrition is essential for promoting lung development and minimizing the risk of pulmonary hypertension. To meet these needs, nutritional support must be individualized, considering factors such as gestational age, birth weight, and clinical condition. It is recommended that preterm infants receive a higher caloric intake to support their rapid growth and development. Sufficient protein intake is particularly crucial for muscle development and overall growth, which in turn can positively influence lung health. Omega-3 fatty acids have the potential to enhance endothelial function, which is critical for the regulation of pulmonary vascular tone. Additionally, incorporating antioxidant-rich foods or supplements, such as vitamin E and vitamin C, may help reduce lung injury in preterm infants. Regular assessments and adjustments to the nutritional regimen may be necessary, depending on the infant’s clinical response and condition [8]. This scenario offers an opportunity for early detection and intervention, which can increase survival rates and promote long-term health outcomes [7].

Research shows that the overall outcome for children with pulmonary arterial hypertension is poor due to the pathophysiological mechanisms causing chronic hypoxia and nutritional deficits [9]. Hypoxemia, observed in congenital heart disease, has been demonstrated to be linked to lower levels of endocrine factors, potentially leading to growth failure [10].

A study that followed 601 children (ranging from newborns to 5-year-olds) for a median of 2.9 years (IQR 1.5–4.4) reported the following data: the median baseline height-for-age percentile was 26 (IQR 4–54) and the median baseline body mass index (BMI)-for-age percentile was 41 (IQR 12–79). The Z-score for height-for-age was considerably lower than the reference (−0.81, 95% CI −0.93 to −0.69; *p* < 0.0001), as was the Z-score for BMI-for-age (−0.12, −0.25 to −0.01; *p* = 0.047). The Z-score for height-for-age was mainly low in younger patients (≤5 years) with idiopathic or hereditary pulmonary hypertension and in all patients with pulmonary hypertension associated with congenital heart disease. The extent of impairment was associated with the cause of PAH, comorbidities, and the severity and duration of the disease. As favorable clinical outcomes were associated with compensatory growth, height-for-age could represent an additional globally available clinical parameter for monitoring the clinical status of patients [9].

## 2. Nutrition in Early Childhood

Breastfeeding and human milk are considered the standard benchmarks for infant feeding and nutrition. Considering the proven short- and long-term advantages of breastfeeding for both medical health and neurodevelopment, infant nutrition ought to be regarded as a public health concern rather than just a lifestyle choice. The American Academy of Pediatrics reiterates its recommendation of exclusive breastfeeding for approximately six months, followed by the continuation of breastfeeding as complementary foods are introduced. Breastfeeding should be extended for one year or longer, as mutually desired by both the mother and the infant [10]. Exclusive breastfeeding is defined by the World Health Organization as the practice in which only breast milk is given to the infant during the first six months of life, without any other liquids or foods, except for oral rehydration salts and vitamin and mineral supplements, as well as medications [11,12].

Breastfeeding has also been shown to decrease upper and lower respiratory tract infections (by 72%), middle ear infections (by 23%), and diarrhea in infants [13]. Breast milk coats the nasopharyngeal mucosa, thereby protecting against the transmission of microorganisms responsible for respiratory illnesses [14]. It is also suggested to reduce the risk of asthma (by up to 40%), food allergies, sudden infant death syndrome (SIDS) by 30%, inflammatory bowel disease (by 40%), type 2 diabetes (by 40%), and obesity [13]. This is linked to a 64% reduction in the occurrence of nonspecific gastrointestinal infections, and this effect remains for two months following the weaning of the infant [15].

Providing breast milk to preterm infants offers substantial benefits both in the short and long term. The reduced rates of sepsis and necrotizing enterocolitis suggest that human milk plays a key role in promoting the development of underdeveloped immune defenses in premature infants [16]. The various elements of breast milk establish the biological basis for its protective benefits against gastrointestinal and respiratory illnesses. It comprises an array of bioactive factors, including antibodies (like Immunoglobulin A), prebiotic oligosaccharides, lactoferrin, nonspecific anti-infective substances, leukocytes, lymphocytes, probiotics, and other immune cells, as well as beneficial microbes. These substances work together to support the infant’s immune system and safeguard the child against infections [17]. Studies indicate that IgA hinders viruses and bacteria from binding to mucosal epithelial cells, which may result in infections [18].

The primary factors contributing to the discontinuation of breastfeeding are a reduction in milk supply, an improper latch, and soreness of the breasts or nipples [19].

As solid foods are introduced, diet becomes crucial for regulating the structure and role of the microbiota. Several studies have explored the impact of major nutrients—fats, carbohydrates, and proteins—on the gastrointestinal microbiome. Furthermore, other elements of the diet, such as soluble and insoluble fibers, also contribute substantially to shaping the health of the microbiota [20,21,22,23]. Many dietary components act as compounds utilized by bacterial enzymes, leading to enzymatic processes that produce byproducts that can be absorbed by the intestines. Notably, butyric and acetic acids, primarily derived from the bacterial breakdown of fibers, are recognized for promoting circulatory system health. On the other hand, trimethylamine-N-oxide, a byproduct produced by gut microbes from carnitine, betaine and choline, abundantly found in meat, eggs, and fish—is associated with an increased risk of cardiovascular disease [21].

**Figure 1:**
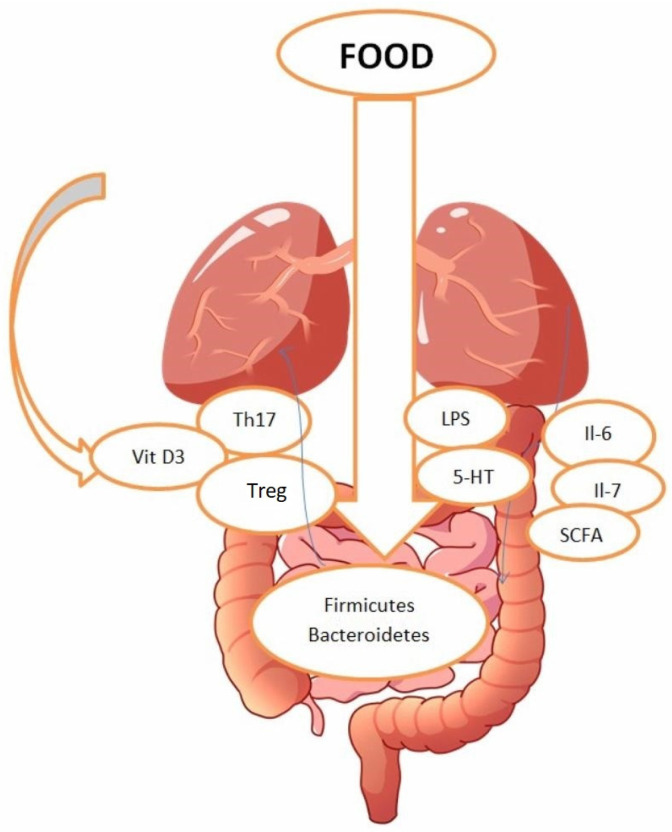
Influence of nutrition on PAH: The role of dietary factors such as Fe^2+^, vitamins D and C, flavonoids, and other related polyphenols, alongside vitamin D acquired from sunlight exposure, may enhance the quality of life and outcomes of patients suffering from pulmonary arterial hypertension. Each nutritional element operates through its own unique mechanism. Nevertheless, certain effects of these nutrients could be linked to their impact on the immune system via the rejuvenation of T lymphocytes and cytokines, alterations in the gut microbiome and its bacterial metabolites, and the movement of bacteria across barriers [25].

Patients experiencing pulmonary hypertension and malabsorption need to be attentive about what they are eating. It is essential to address both their nutritional needs and the challenges associated with nutrient absorption. Their diet should include high-calorie, high-protein foods and those that are rich in fats, and proteins that are easier to digest (like blended foods or those that require less digestive effort) can be more suitable for PH patients with malabsorption. Micronutrient supplementation (including supplementation of zinc, magnesium, and calcium) is also recommended. For patients with severe malabsorption, enzyme replacement therapy may be necessary to assist in the digestion and absorption of fats, proteins, and carbohydrates, ensuring adequate nutrient intake [24]. Multiple factors are involved in pulmonary hypertension and one of them is nutrition. The role of nutrition in influencing pulmonary hypertension has been established in multiple epidemiological studies conducted on human and animal models; however, the European Society of Cardiology has not provided dietary guidelines for treating patients with PH [1,25]. The importance of nutrition is very broad, playing a key part in the development, progression and management of pulmonary hypertension, especially in children with associated congenital abnormalities. These patients often experience impaired growth and various nutrient deficiencies, which are further associated with factors like malnutrition [26,27]. Various studies have observed that the consequences of malnutrition are negative in children with pulmonary hypertension, suggesting that nutritional status is linked to clinical outcomes and that it can serve as a useful predictive tool [28]. Also, obesity is taken into account as a risk factor for PH [29]. According to a specific study, maternal obesity can lead to pulmonary hypertension in offspring by an interleukin-6 mediated mechanism with a role in smooth muscle cell proliferation [30]. While there are not many studies on this topic that specifically focus on children, we can extrapolate the data from expecting mothers and their relation to overweight status (Figure 1).

At the same time, the effects of various nutrients are being studied for their influence on pediatric pulmonary hypertension. Severe vitamin C deficiency may contribute to pulmonary hypertension, though its exact role is not fully understood. It has been proven to affect nitric oxide signaling, which could be the cause [31]. On the other hand, the role of vitamin D in cardiovascular diseases seems to be better understood. It is implicated in many fundamental mechanisms, such as oxidative stress and cell proliferation, that could affect children [32].

These are but a few of the nutritional factors that contribute to influencing pediatric pulmonary hypertension, which will be detailed in this review. Thus, we aim to focus on how such elements might affect the development or progression of PH. Additionally, we will cover important information about nutrition in early childhood. Another important subject will be the significance of pulmonary hypertension on child outcome and child growth. Last but not least, we intend to explore the topic of promising nutrients for treating cardiovascular diseases, such as resveratrol, whose properties facilitate the inhibition of oxidative, inflammatory, proliferative, and fibrotic processes [33].

Taking this into consideration, we can turn our attention to important factors that influence this pathology.

## 3. Malnutrition Associated with PH

**Figure 2:**
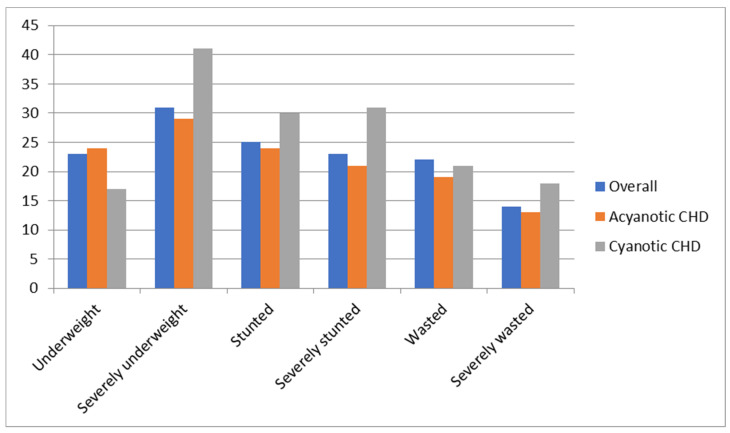
Incidence of undernutrition in pediatric patients with CHD. This chart shows the percentage and severity of undernutrition in congenital heart diseases. As shown, at the pinnacle of the chart are pediatric patients with cyanotic CHD, with more than 40% being severely underweight [35].

Malnutrition presents an increased health concern in pediatric patients with PH. In research carried out by Crowell et al., it was found that 51/196 (26%) children with pulmonary hypertension were also diagnosed with malnutrition (Figure 2), though previously, only 5% had an ICD-10 code for nutritional deficiency listed in their medical charts [34].

The incidence of malnutrition in early childhood depends on several factors. Increased energy intake in response to the metabolic stress associated with the malabsorption of nutrients caused by nausea and gastroenterological problems plays a role in causing malnutrition. Additionally, the side effects of medication and fatigue during feeding contribute to reduced nutrient intake [35,36].

Once malnutrition takes hold, it will worsen the outcomes of the pediatric patient. Studies show that it is associated with greater length of ventilation in children admitted to the ICU according to the multivariable logistic regression model (*p* = 0.024, OR = 1.76, 95% CI: 1.08–2.88) [37]. Malnutrition, specifically undernutrition, leads to a reduction in diaphragmatic muscle mass, diminished respiratory muscle strength, lower inspiratory muscle pressure, and increased chest wall resistance, all of which negatively impact lung tissue, musculature, functionality and respiratory dynamics. Another study on kids ranging from 0 to 18 years of age showed that underweight and stunted children showed an association with increased mortality, with an OR = 3.54 (95% CI: 1.62–7.74, *p* < 0.001) and OR of 3.31 (95% CI: 1.65–6.64, *p* < 0.001) [38,39].

## 4. Vitamin C

Vitamin C, also known as L-ascorbic acid, is a water-soluble nutrient that is naturally present in specific foods, added to others, and is available as a dietary supplement. Since people, unlike most animals, cannot synthesize vitamin C internally, it must be obtained through the diet, making it an essential nutrient [40].

Vitamin C is necessary for the functionality of multiple enzymes involved in the renewal of the tissue, is important for the proper functioning of the immune system, and acts as an antioxidant [41]. A great number of studies have shown that oxidative stress is implicated in cardiovascular diseases. Meanwhile, the oxidation of low-density lipoproteins in the endothelial wall determines these particles to be more atherogenic, enabling them to gather in the walls of arteries [42]. Also, experimental studies have shown that oxidative stress impacts endothelial activity in coronary vessels. It plays a role in the production of type IV collagen, which is essential for the formation of the basement membrane and endothelial cell attachment. Additionally, vitamin C is involved in nitric oxide (NO) synthesis and may boost prostacyclin levels. Each of these factors plays a role in the development of pulmonary hypertension [43].

A clinical case has linked scurvy to evolution towards pulmonary hypertension; however, it was established that supplementation with vitamin C reversed the effects [44]. Another similar case described the impact of vitamin C deficiency in an autistic boy who developed pulmonary hypertension and refused to walk. When he arrived, he was lethargic and breathless. Echocardiography revealed a hypertensive right ventricle. His history showed a limited diet lacking in vegetables and including very few fruits. After the initiation of treatment with high-dose vitamin C, he was able to walk again, and his pulmonary pressure returned to normal [45].

From these studies, we can argue that vitamin C should be investigated in pediatric cases with pulmonary hypertension, especially in the ones with poor diets.

## 5. Vitamin D

To become 1,25-hydroxyvitamin D (25(OH)D), vitamin D must be biologically inactive and it requires activation through a two-step hydroxylation process. Additionally, 25(OH)D is an intermediate product, and vitamin D levels are usually assessed by measuring the vitamin D concentration in the human organism [46].

Callejo et al. explained that a lack of vitamin D can reduce nitric oxide-dependent cGMP production, which also suggests a benefit of vitamin D supplementation in patients with pulmonary hypertension [47].

The partial lack of vitamin D has been associated with increased cardiovascular mortality [48]. Thus, it seems necessary for serum levels of vitamin D to be regularly evaluated in patients with this condition. External addition of vitamin D should be used to avoid bone diseases in any individual with moderate or severe deficiency. It is uncertain whether replenishing vitamin D levels enhances the clinical presentation, quality of life, and outlook of patients with pulmonary arterial hypertension (PAH). Other conditions require the use of vitamin D supplementation [25]. A recent meta-analysis confirmed that vitamin D supplementation had no effect on cardiovascular diseases or the risk of type 2 diabetes. Additionally, no clear conclusion was reached regarding the clinical significance of vitamin D supplements in the treatment of hypertension (HTN). A clinical observation suggested that vitamin D deficiency affected the efficacy of sildenafil in treating the condition and restoring vitamin D levels improved this symptom [49].

Yadav and Shah (2023) demonstrated that a combination of vitamin D and sildenafil had a significantly greater effect than vitamin D or sildenafil administered alone when it came to treating pulmonary arterial hypertension (PAH). However, this protective effect was diminished when the combination was given with L-NAME (20 mg/kg). Additionally, administration of MCT resulted in reduced expression of eNOS, an effect that was significantly reversed by a combination of vitamin D and sildenafil compared to monotherapy with either treatment. The efficacy of vitamin D is increased when it is administered alongside sildenafil. Furthermore, vitamin D deficiency in the context of pulmonary hypertension is linked to increased severity of PAH. Consequently, the combination of sildenafil and vitamin D may produce a synergistic effect, contributing to more effective management of this condition [50].

## 6. Iron Deficiency

Iron has a key role in various physiological mechanisms, such as oxygen delivery and energy metabolism. Approximately 70% of the body’s iron is bound to hemoglobin, while about 5–10% is found within myoglobin. The most precise laboratory parameter used to assess iron reserves is serum ferritin [51]. In accordance with the 2022 guidelines from the European Society of Cardiology (ESC) and the European Respiratory Society (ERS), iron deficiency is defined similarly to its definition in heart failure, characterized by ferritin levels of less than 100 µg/mL or between 100 and 299 ng/mL, along with transferrin saturation (TSAT) of less than 20% [1]. Iron is an essential micronutrient with multiple roles in the cardiovascular system, one of which is its critical participation in oxygen transport as a component of hemoglobin. In cases of pulmonary hypertension, iron deficiency can impair oxygen delivery, worsening the oxygenation compromise experienced by patients. Additionally, iron deficiency can disrupt cellular energy metabolism, resulting in cellular hypoxia and oxidative stress [52]. Fairly recently, Lakhal-Littleton et al. demonstrated that in mice, intracellular iron insufficiency in pulmonary arterial smooth muscle cells (PASMC) leads to pulmonary arterial hypertension (PAH) by increasing the expression of ET-1 [53]. Howard and colleagues conducted a thorough epidemiological analysis that revealed iron deficiency is a commonly encountered comorbidity in patients with pulmonary hypertension, occurring even without the presence of anemia [54].

A study conducted by Ruiter et al. on children from 12 years old to 18 years old indicated that oral iron therapy effectively increased ferritin levels in only 8 (44%) of the 18 patients, indicating that absorption of iron in the system may be dysfunctional in these individuals [55].

Research has shown that the depletion of iron can inhibit the progression of right ventricular hypertrophy in pulmonary hypertension [56].

Breastfed infants will receive all their nutritional value from their mother’s milk during the first six months after birth. If the mothers cannot breastfeed, iron-fortified infant formula purchased from the store should be used for the first 9 to 12 months. Following that, a cow’s milk-based formula can be introduced [57].

Regarding diversification, it is important to consider several categories: (a) meats such as chicken, lamb, veal, pork, liver, beef, and turkey; (b) aquatic animals, noting that it is essential for infants under 1 year to avoid shellfish, such as lobster, shrimp, and clams, to reduce the risk of allergic reactions; (c) eggs: for infants under 1 year, it is recommended to avoid egg whites, as they may present an allergy risk; (d) cereals and fats: cereals, whole grain bread, bread, pasta, and rice with additional iron are excellent sources and can be easily included in an infant’s diet; (e) legumes: chickpeas, lentils, dried peas, and beans are all rich in iron; (f) vegetables, including beans, brussels sprouts, broccoli, green peas, and spinach, also provide iron and additional nutrition [58].

## 7. Resveratrol—A Newly Studied Promising Nutrient

**Figure 3:**
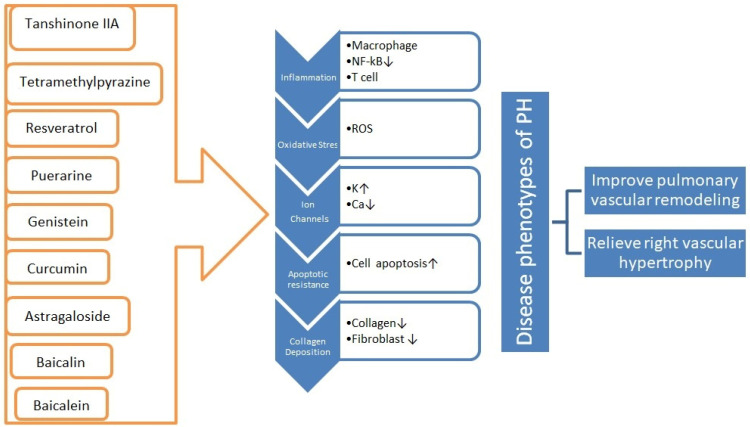
Natural products with benefic role in pulmonary hypertension. These extensively researched natural compounds may contribute to the management of pulmonary hypertension (PH) by enhancing anti-inflammatory responses, suppressing oxidative stress, mitigating apoptosis resistance, and modulating dysregulated ion channels and collagen accumulation. These mechanisms can effectively alleviate pulmonary vascular remodeling and right ventricular hypertrophy [59].

There are multiple natural products that have been studied for their role in treating pulmonary hypertension by inhibiting oxidative stress, improving inflammatory responses, regulating abnormal ion channels, and reducing apoptotic resistance and collagen deposition (Figure 3). One such natural product is resveratrol [59].

Resveratrol, also known as 3,5,4′-trihydroxystilbene, is a nutritional substance with remakable oxidative stress-reducing, inflammation-reducing, and endothelial-shielding effects in the systemic circulation. There are studies on rodents that hypothesize its role in providing antiproliferative and vasoprotective effects [60]. In a study on rats, it was established that it inhibits right ventricular myocyte hypertrophy after chronic hypoxia (*p* < 0.01), lowers right ventricular systolic pressure, and reduces neutrophil infiltration in the lungs [61]. On the other hand, using resveratrol via oral or intravenous routes may lead to side effects such as liver damage, nausea, diarrhea, and breathlessness. Inhaling resveratrol microparticles might be an alternative for increasing bioavailability and easing administration [62].

Finally, a systematic review of 1724 articles on both adults and children concluded that, even in low doses (10–100 micromol/L), resveratrol presents protective heart actions, reduces inflammation and oxidation, as well as having chemoprotective characteristics [63].

## 8. Microbiome

The human gut microbiome (GM) is believed to include approximately 10^14^ microorganisms, primarily anaerobic bacteria. It plays a crucial role in various biochemical functions, especially in maintaining gastrointestinal mucosal stability, synthesizing vitamins, supporting the immune system, metabolizing drugs, and regulating energy production. While each individual’s gut microbiome is one of a kind, it can be influenced by outside factors such as lifestyle, diet, and drugs, especially antibiotics [64].

Infancy is increasingly recognized as a critical period for microbiome establishment, which influences both neonatal health and long-term adult outcomes. For instance, early microbiome alterations have been correlated with later development of diseases such as type 1 diabetes, obesity, and asthma. This emphasizes the potential role of the microbiome in shaping early health trajectories and contributing to overall development [65].

Multiple studies were conducted on rats to determine if there is a connection between gut dysbiosis and pulmonary arterial hypertension. A relevant study on the adult population that can be extrapolated to pediatric patients showed that gut dysbiosis can lead to several immune dysbalances, which have been implicated in the mechanisms of pulmonary arterial hypertension. Notably, increased TLR4 activation has been observed in PAH. Additionally, gut dysbiosis has been linked to the activation of Th1 and Th17 CD4 cells, elevated IL-17 secretion, and the downregulation of Tregs. Another important fact is that the activation of Th1 and Th17 CD4 cells, along with Treg dysfunction, has been specifically associated with the development of PAH. These findings suggest that gut disturbances may take part in initiating immune imbalance and early inflammation around blood vessels in PAH [66].

Another type of microbiome that is particularly important for patients, especially in children, is the airway/lung microbiome. A recent study conducted on 156 children revealed lower microbial richness in the airway microbiome of those with congenital heart disease, along with an increased presence of Streptococcus and Rothia [67].

## 9. Discussion

As mentioned at the beginning of the article, pulmonary hypertension is a severe diagnosis, especially for a newborn. In most cases, the associated diseases are part of the cardiological spectrum (Eisenmenger syndrome, uncorrected septal defects, cardiomyopathies, transposition of the great arteries, etc.). In some cases, by treating the underlying pathology in time, we can prevent the development of pulmonary hypertension.

In our study, we also monitored the necessary nutrition for patients at risk of the condition, as due to the pathophysiological mechanisms, deficiencies of iron (with or without the presence of anemia) and vitamins, especially fat-soluble ones (C, D), as well as polyphenolic compounds (resveratrol), may occur. All cited articles supported the necessity of treating these deficiencies to improve the quality of life of the children.

Vitamin D has been shown to have a synergistic effect with 5-phosphodiesterase inhibitors, enhancing their effect. Meanwhile, vitamin C, which plays a role in tissue repair and acts as an antioxidant, contributes to nitric oxide synthesis, thus regulating mechanisms altered in the development of pulmonary hypertension.

Iron deficiency can occur with or without the presence of anemia, as it is not dependent on this factor. We know that iron plays an important role in oxygen transport within the body, and its deficiency could amplify the cyanosis present in this pathology.

The studies we reviewed supported a deficit in the growth of children with pulmonary hypertension compared to those who are medically healthy. Thus, when it comes to the nutrition of these children, they rely on breastfeeding for the first 6 months, and later, when solid foods are introduced to their diet, it is important to provide foods rich in the deficient vitamins and elements, such as meat, eggs, vegetables, and legumes, to ensure proper development.

Given the complexity of nutrition in relation to the presented pathology, in future, researchers may wish to study the diets of young children to ensure that the symptoms of pulmonary hypertension are minimal to absent. In our study, we emphasized the importance of vitamins, but the optimal quantity of each and the most efficient source to meet the mentioned needs have yet to be determined.

## 10. Limitations of the Study

This review presents several limitations that must be considered. First, the availability of research focusing specifically on pediatric pulmonary hypertension (PH) is limited, particularly in developing countries, where the prevalence of the disease may differ from that in developed regions. The majority of clinical trials and data have been extrapolated from adult populations, which could limit these findings’ accuracy and relevance to children. Additionally, many of the studies discussed rely on animal models, and the translation of these results to human pediatric populations is not always straightforward.

Moreover, our review identifies a range of nutritional factors that may influence PH, yet there is a deficiency of large-scale, longitudinal studies assessing the direct impact of these nutrients on pediatric PH outcomes. This gap highlights the need for more targeted research that includes randomized controlled trials focused on nutritional interventions in children. Finally, the role of socio-economic and environmental factors, which could significantly affect both nutrition and health outcomes in pediatric PH, is not comprehensively addressed in this review.

## 11. Conclusions

This review underscores the critical implication of nutrition in the development, progression, and management of pediatric pulmonary hypertension (PH). While many of the data are extrapolated from adult populations or animal models, emerging evidence suggests that nutritional insufficiencies, such as those of vitamins C, D, and iron, play an important role in exacerbating PH in children. The importance of early childhood nutrition, particularly in preventing malnutrition and promoting healthy growth, is highlighted as a promising route for improving clinical outcomes in pediatric patients.

Promising nutrients like resveratrol and the potential modulation of the gut and respiratory microbiome also show therapeutic potential, though further research is needed to establish their efficacy in pediatric populations. The review emphasizes the need for targeted, pediatric-specific research to develop effective nutritional guidelines that could aid in the treatment and prevention of PH. Ultimately, optimizing nutritional interventions might act as an important resource for the management of pediatric PH and for improving long-term outcomes for affected children.

## Data Availability

Data sharing not applicable.
